# Quantification of the relative contribution of the different right ventricular wall motion components to right ventricular ejection fraction: the ReVISION method

**DOI:** 10.1186/s12947-017-0100-0

**Published:** 2017-03-27

**Authors:** Bálint Lakatos, Zoltán Tősér, Márton Tokodi, Alexandra Doronina, Annamária Kosztin, Denisa Muraru, Luigi P. Badano, Attila Kovács, Béla Merkely

**Affiliations:** 1 MTA-SE Cardiovascular Imaging Research Group, Heart and Vascular Center, Semmelweis University, Városmajor St. 68, H-1122 Budapest, Hungary; 2 Department of Software Technology and Methodology, Eötvös Loránd University, Budapest, Hungary; 3 Department of Cardiac, Thoracic and Vascular Sciences, University of Padova, Padova, Italy

**Keywords:** 3D echocardiography, Right ventricle, Decomposed wall motion

## Abstract

Three major mechanisms contribute to right ventricular (RV) pump function: (i) shortening of the longitudinal axis with traction of the tricuspid annulus towards the apex; (ii) inward movement of the RV free wall; (iii) bulging of the interventricular septum into the RV and stretching the free wall over the septum. The relative contribution of the aforementioned mechanisms to RV pump function may change in different pathological conditions.

Our aim was to develop a custom method to separately assess the extent of longitudinal, radial and anteroposterior displacement of the RV walls and to quantify their relative contribution to global RV ejection fraction using 3D data sets obtained by echocardiography.

Accordingly, we decomposed the movement of the exported RV beutel wall in a vertex based manner. The volumes of the beutels accounting for the RV wall motion in only one direction (either longitudinal, radial, or anteroposterior) were calculated at each time frame using the signed tetrahedron method. Then, the relative contribution of the RV wall motion along the three different directions to global RV ejection fraction was calculated either as the ratio of the given direction’s ejection fraction to global ejection fraction and as the frame-by-frame RV volume change (∆V/∆t) along the three motion directions.

The ReVISION (Right VentrIcular Separate wall motIon quantificatiON) method may contribute to a better understanding of the pathophysiology of RV mechanical adaptations to different loading conditions and diseases.

**Electronic supplementary material:**

The online version of this article (doi:10.1186/s12947-017-0100-0) contains supplementary material, which is available to authorized users.

## Introduction

During the last decades the right side of the heart has gained more and more attention. Compared to the relatively simple conical shape of the left ventricle, the right ventricle (RV) shows a more complex anatomical structure. When viewed from anterior-lateral view it shows a triangular shape, whereas when it is viewed from a short axis cross-section it has a crescent shape, partially wrapping the left ventricle. Anatomically, the RV is traditionally divided into three parts: the inlet portion, which consists of the tricuspid valve, chordae tendinae and the papillary muscles, the apical part with trabeculated muscle, and the outlet portion, also called the infundibulum with the pulmonary valve, which seperates the ventricle from the pulmonary trunk [1]. In contrast with the predominantly oblique arrangement of the left ventricular myocytes, the RV free wall has two layers: in the subepicardium the myocardial fibres are oriented circumferentially, whereas in the subendocardium they are directed longitudinally [1]. Finally, the RV shows a distinctive, peristaltic-like contraction pattern: the activation starts from the inlet portion and ends at the outlet [2].

Three main mechanisms contribute to RV pump function: (i) shortening of the longitudinal axis with traction of the tricuspid annulus towards the apex; (ii) inward movement of the RV free wall; (iii) bulging of the interventricular septum into the RV during the left ventricular contraction and stretching the free wall of the RV over the septum [3]. Since the relative contribution of the three mechanism may vary in different cardiac conditions [4], the availability of a technique able to assess the relative contribution of these three components to the global RV pump function will help to clarify the pathophysiology and the mechanical adaptations of the RV in different loading conditions.

### Shortcomings of current imaging techniques in the evaluation of the RV

The complexity of RV geometry and mechanics may in part explain current difficulties in the assessment of its function using conventional tomographic techniques. The most widely used two-dimensional transthoracic echocardiography provides several parameters, however, the majority of the conventional measures refer only to the longitudinal contraction of the chamber [5]. Three-dimensional (3D) echocardiography offers a unique opportunity to map the whole endocardial surface of the RV independent on any assumption about its shape and to display its motion using surface rendering modalities [6]. Since the RV pump function is the result of several different mechanisms, just measuring the RV ejection fraction may not be sensitive enough to characterize the different pathological conditions. The relative contribution of the three aforementioned mechanisms to global RV function may be different in certain diseases affecting the RV and therefore, being able to obtain a separate quantification of the relative contribution of each of them could hold diagnostic and prognostic information.

Accordingly, our aim was to develop a custom method to separately quantify the extent of longitudinal, radial and anteroposterior displacement of the RV walls and their relative contribution to the global RV ejection fraction using 3D data sets of the RV obtained by echocardiography.

## Material and methods

### Image acquisition and 3D model reconstruction

Current matrix-array transducers permit the acquisition of 3D volumetric data of the RV during routine transthoracic echocardiographic examinations [7]. Dedicated software (4D RV-Function 2, TomTec Imaging GmbH, Unterschleissheim, Germany) is commercially available to generate a 3D surface rendering model (beutel) of the RV by a semi-automated algorithm [8, 9]. Time series of this 3D model (i.e. series of polygon meshes) can be exported volume-by-volume throughout the cardiac cycle (Additional file 1).

### Novel method for decomposing the motion of the RV

**Fig. 1:**
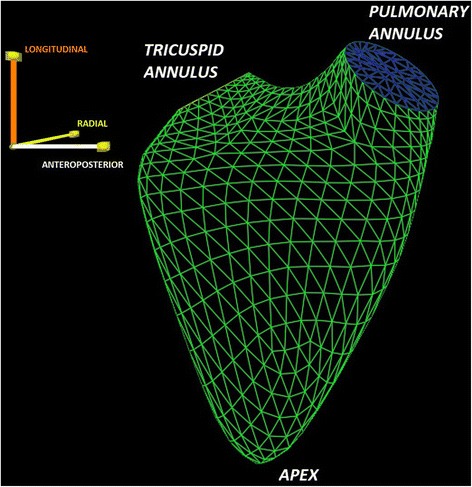
Example of the exported mesh (*right ventricular beutel*) using the wireframe surface rendering display method. The model is positioned to correspond to the three anatomically relevant axes (longitudinal, radial and anteroposterior)

**Fig. 2:**
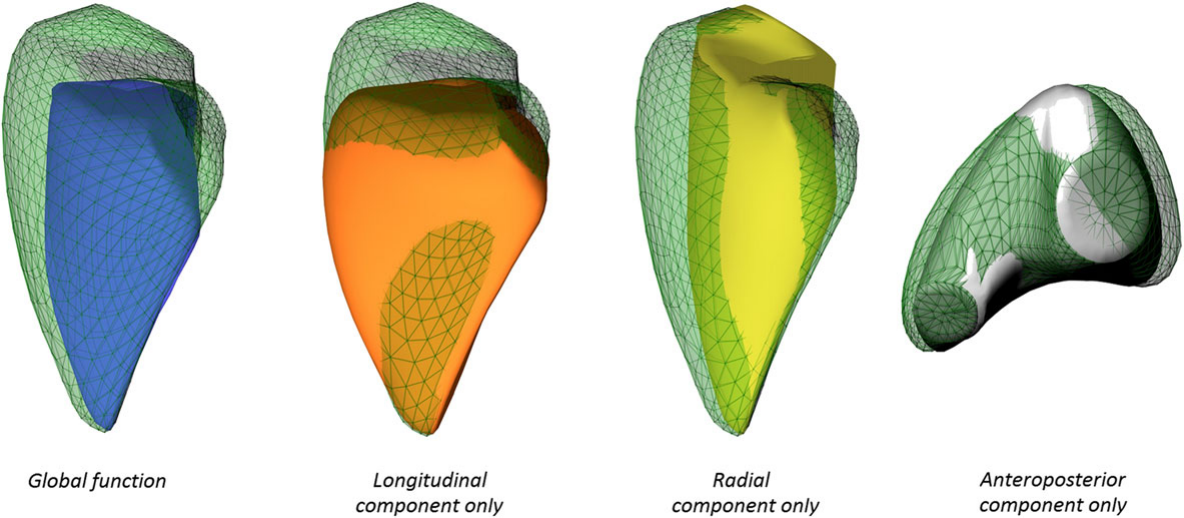
The motion of the right ventricular wall during the cardiac cycle can be decomposed along three anatomically relevant axes (longitudinal, radial and anteroposterior) and the change of the volume during the cardiac cycle can be measured for each axis

We developed the ReVISION (Right VentrIcular Separate wall motIon quantificatiON) method using the Unity3D engine to decompose the motion of the exported RV beutel along three orthogonal axes and calculate the respective volume at each time frame. Note that the Euclidean axes in the dedicated software’s output correspond to the anatomically relevant ones (longitudinal, radial and anteroposterior). We decomposed the movement of the RV wall in a vertex-based manner (e.g. for the longitudinal motion we took into account only the movement of the vertices along the Y axis) (Figs. 1 and 2; Additional files 2, 3 and 4). That is for each m_1_,…, m_n_ series of meshes where ${m}_i=\left\{\left({x}_1^i,{y}_1^i,{z}_1^i\right),\dots, \left({x}_k^i,{y}_k^i,{z}_k^i\right)\right\}$



**Additional file 1:** Right ventricular model with all motion directions enabled (Animation 1 – global.mp4). (MP4 468 kb)




**Additional file 2:** Right ventricular model with only longitudinal motion enabled (Animation 2 – longitudinal only.mp4). (MP4 486 kb)




**Additional file 3:** Right ventricular model with only radial motion enabled (Animation 3 – radial only.mp4). (MP4 426 kb)




**Additional file 4:** Right ventricular model with only anteroposterior motion enabled (Animation 4 – anteroposterior only.mp4). (MP4 728 kb)

$$ {m}_i^y:=\left\{\left({x}_1^i,{y}_j^i,{z}_1^i\right)\Big|\left({x}_j^i,{y}_j^i,{z}_j^i\right)\in {m}_i\right\} $$

**Fig. 3:**
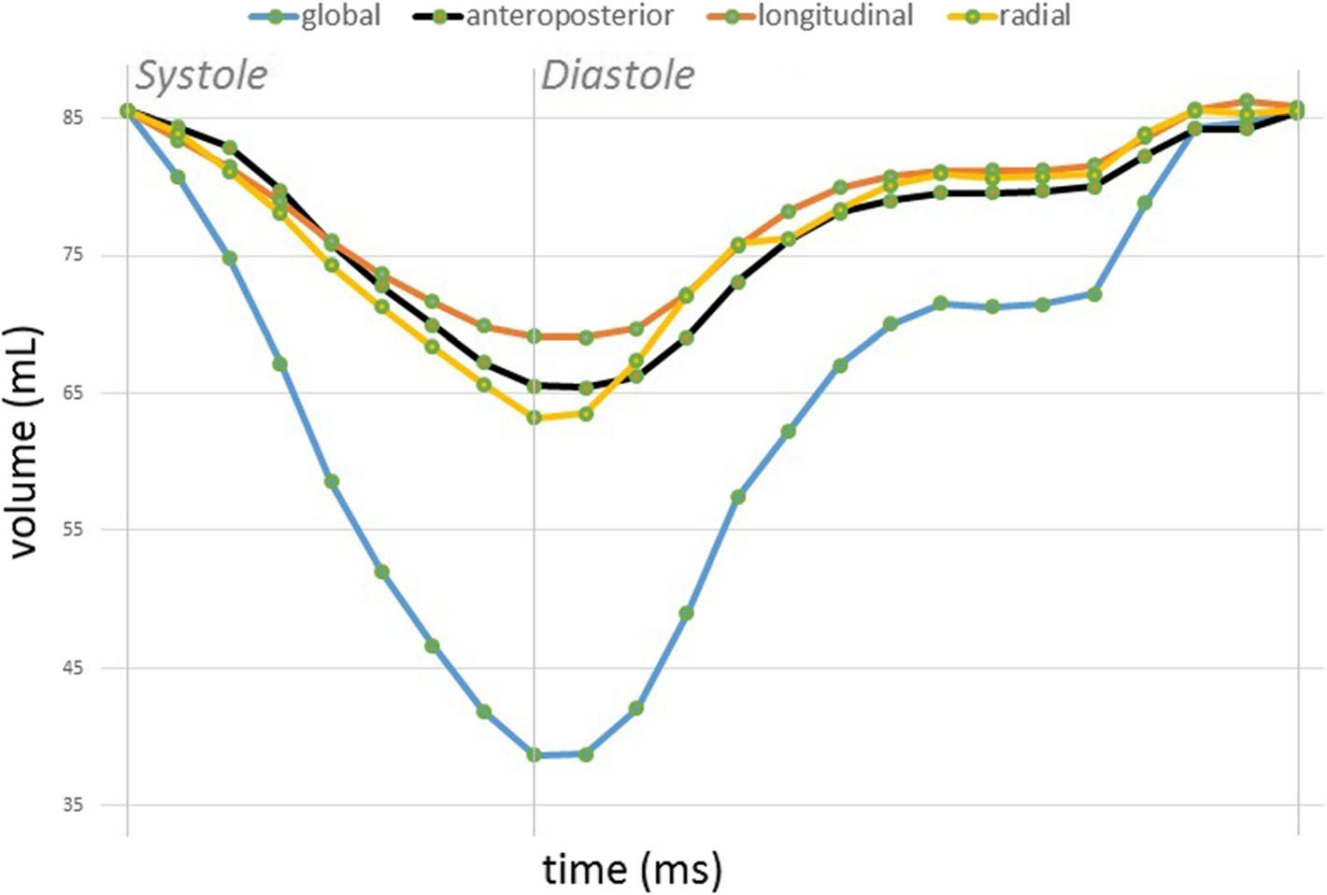
One beat global (*blue line*) and decomposed volume-time curves of the right ventricle in a healthy volunteer

The volumes of the beutels accounting for the RV wall motion in only one direction (either longitudinal, radial, or anteroposterior) were calculated at each time frame using the signed tetrahedron method [10] (Fig. 3). No triangulation step was necessary, as all the polygons of the RV beutel were already triangles.

Let *P* be the set triangles of an *m*_*i*_^*y*^ mesh. For each *p* ∈ *P* let *p* = {(*x*_1,_ *y*_1_, *z*_1_), …, (*x*_3,_ *y*_3_, *z*_3_)} The volume of tetrahedron bounded by the vertices of *p* and the origin:

$$ {V}_p:=\frac{1}{6}\left(-{x}_3{y}_2{z}_1+{x}_2{y}_3{z}_1+{x}_3{y}_1{z}_2-{x}_1{y}_3{z}_2-{x}_2{y}_1{z}_3+{x}_1{y}_2{z}_3\right) $$

Note that *V*_*p*_ is signed, meaning that the values may be negative in case the normal vector of the polygon points towards the origin (when the dot product of a position vector of one of its vertices and the normal vector of the polygon is negative). The volume of the mesh is the sum of the signed *V*_*p*_ volumes:

$$ V = {\displaystyle \sum_{p\in P}}{V}_p $$

### Novel parameters of RV function

**Fig. 4:**
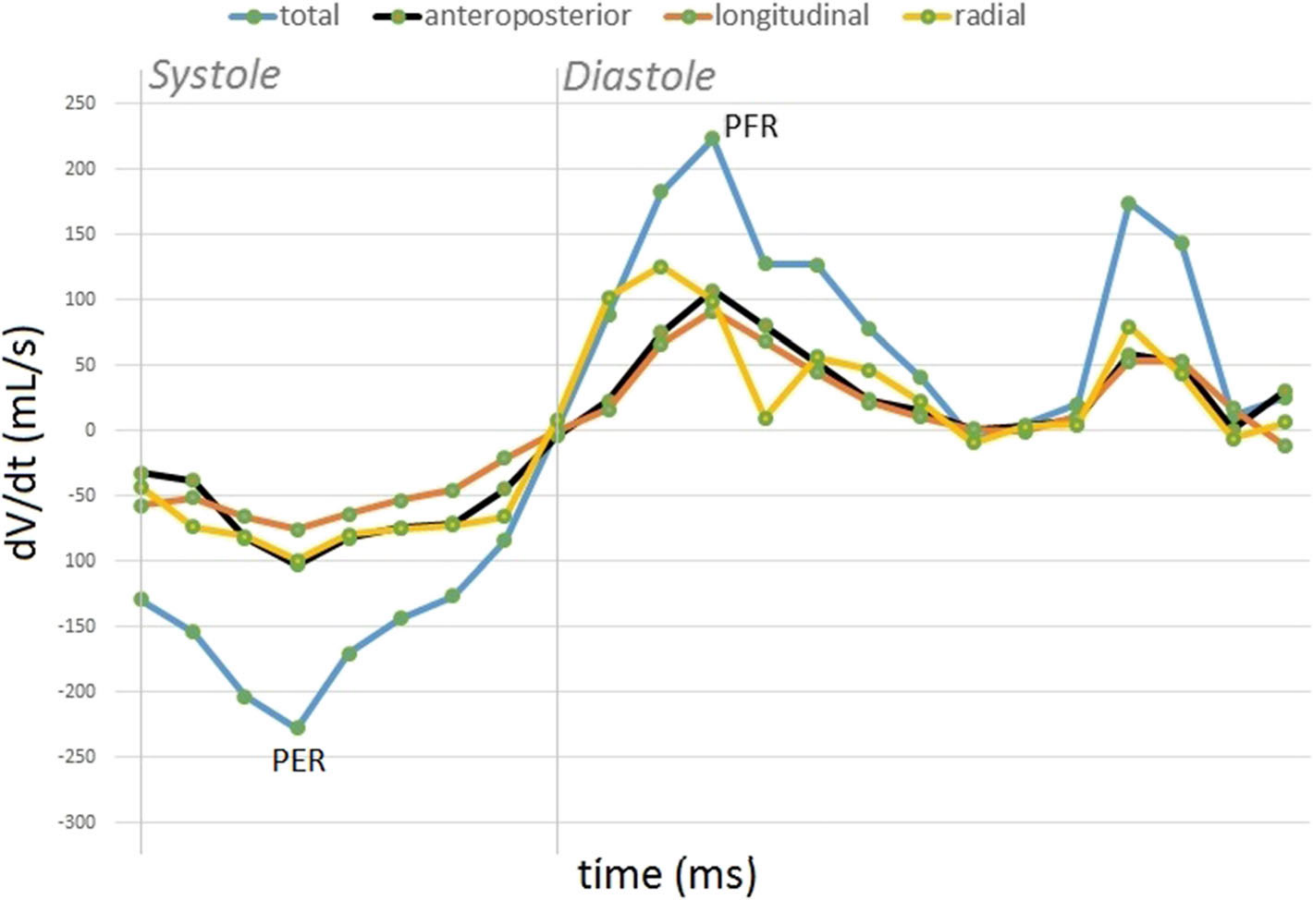
Global (*blue line*) and decomposed dV/dt curves of the right ventricle in a healthy volunteer. PER – Peak Ejection Rate; PFR: Peak Filling Rate

Ejection fraction is the most common measure of RV pump function and it is defined as the difference between end-diastolic and end-systolic volumes indexed to end-diastolic volume. Using the ReVISION method, volume changes due to the RV wall motion along the three directions can be separately quantified and the corresponding ejection fraction value can be calculated (i.e. radial ejection fraction). The relative contribution of the RV wall motion along the three different directions to global RV ejection fraction can be expressed by the ratio of the given direction’s ejection fraction to global ejection fraction. It may be also clinically relevant to evaluate the frame-by-frame RV volume change along the three motion directions. By calculating the difference quotients between the given frames (∆V/∆t), we were able to estimate the relative contribution of longitudinal, radial and anteroposterior motion to systolic, and also to diastolic RV function (Fig. 4). It is important to note, that these parameters are measured in a completely automated way, therefore, the ReVISION method implies no intra- or interobserver variability.

## Discussion

We have shown that, using surface rendered data sets of the RV obtained with transthoracic 3D echocardiography, it is possible to decompose the displacement of the RV wall in a vertex-based manner. The volumes of the beutels accounting for the RV wall motion in only one direction (either longitudinal, radial, or anteroposterior) can be calculated at each time frame using the signed tetrahedron method. Using the ReVISION, we can compute novel parameters of RV function (the relative contribution of the different RV wall motions to global RV ejection fraction and the frame-by-frame RV volume change (∆V/∆t) along the three aforementioned directions). These parameters may help to improve our understanding of the pathophysiology of RV mechanical adaptations to different loading conditions and diseases (Table 1).

**Table 1:** Potential clinical applications of the ReVISION method

Scenarios of clinical interest and suggested functional alterations	
Healthy subjects	Normal contribution of the three components
Post-cardiac surgery patients	Potential predominance of radial displacement
Heart transplanted patients	Long-term predominance of radial displacement
Pressure overload conditions (pulmonary hypertension, acute pulmonary embolism)	Potential reduction of radial displacement
Volume overload conditions (pulmonary regurgitation, atrial septal defect)	Potential reduction of longitudinal displacement
Athlete’s heart	Effects of regular and acute exhaustive exercise
Right ventricular ischemia	Regional and/or global abnormalities
Arrhythmogenic right ventricular dysplasia	Regional and/or global abnormalities
Congenital heart diseases	Depending on the pathology

The RV is a significant contributor to global heart function and therefore, a better understanding of its functional pattern may be of high clinical interest. Despite the fact that the relative contribution of the longitudinal shortening, radial displacement and septal thickening to global RV function may change in different diseases affecting the RV [5], there is no established method to evaluate their importance to global RV ejection fraction.

### Conventional echocardiographic parameters of the RV

**Fig. 5:**
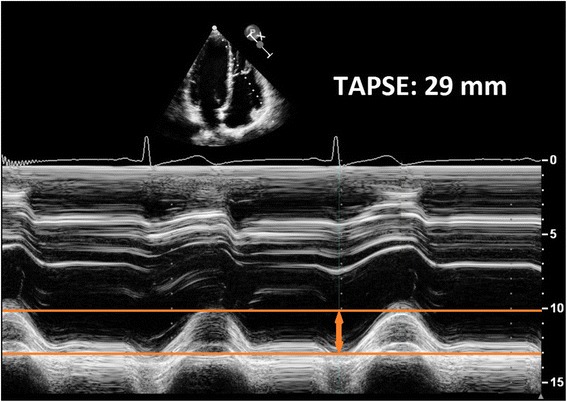
M-mode tracing of the Tricupid Annular Plane Systolic Excursion (TAPSE). Note that this parameter reflects only the longitudinal motion of the right ventricle

**Fig. 6:**
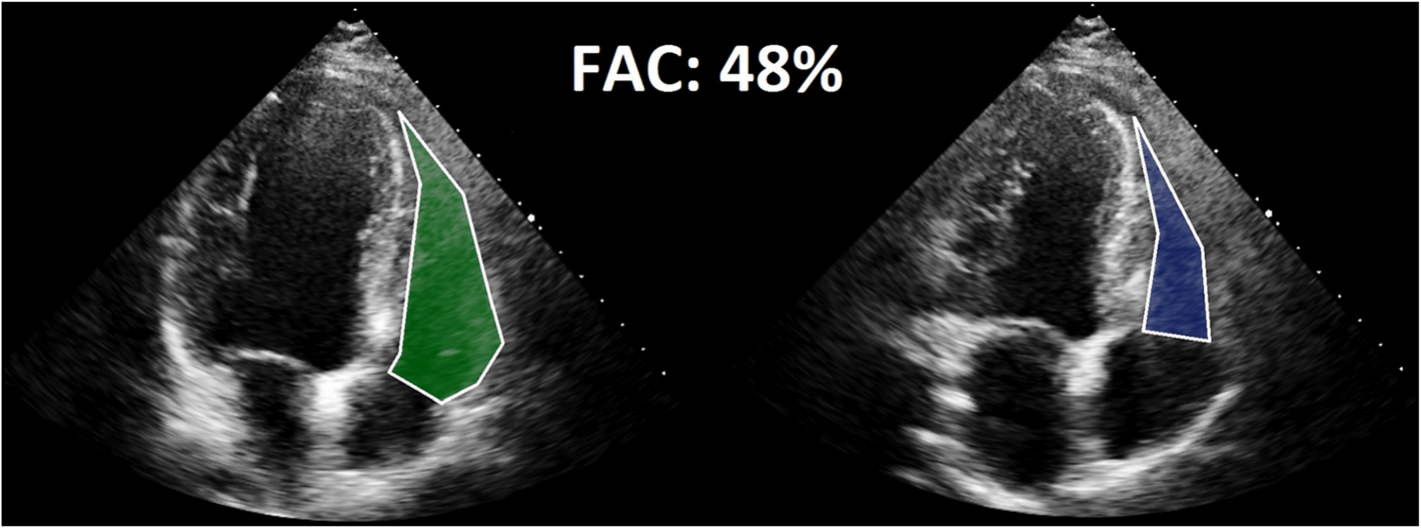
Fractional Area Change (FAC) measurement of the right ventricle

Echocardiography is a widely used, non-invasive imaging modality to describe cardiac morphology and function. However, ultrasonic assessment of the RV is challenging due to the complex anatomy of the chamber. Conventional two-dimensional acquisition protocols require to obtain several imaging views using the parasternal, apical and subcostal approaches. The most commonly used echocardiographic measures to characterize RV morphology are simple linear diameters, while functional aspects are mainly investigated by measuring the tricuspid annular plane systolic excursion (TAPSE) and the RV fractional area change (FAC) [11]. The latter parameters have shown their correlation with RV ejection fraction measured by cardiac magnetic resonance imaging (MRI) [12] and proven their prognostic value as well [13–16]. Nevertheless, several limitations have to be taken into consideration, especially in certain pathological conditions. TAPSE is an easy-to-obtain M-mode parameter of RV function, referring solely to longitudinal motion of RV free wall (Fig. 5). However, since the reference is outside of the heart, TAPSE measures not only the shortening of the RV free wall but also the traction of the RV resulting from left ventricular contraction and the effects of the heart translation within the chest [4]. FAC incorporates both the radial displacement of the RV free wall and the longitudinal motion of the tricuspid annulus toward the apex, assessed on a single tomographic apical four-chamber view and therefore, suffers from the inherent limitations of the limited RV myocardial mass included in this measurement (Fig. 6). These shortcomings of TAPSE and FAC can be overcome by the acquisition of a 3D data set including the whole RV using echocardiography.

### Relative contribution of the different mechanisms in certain cardiac conditions

**Fig. 7:**
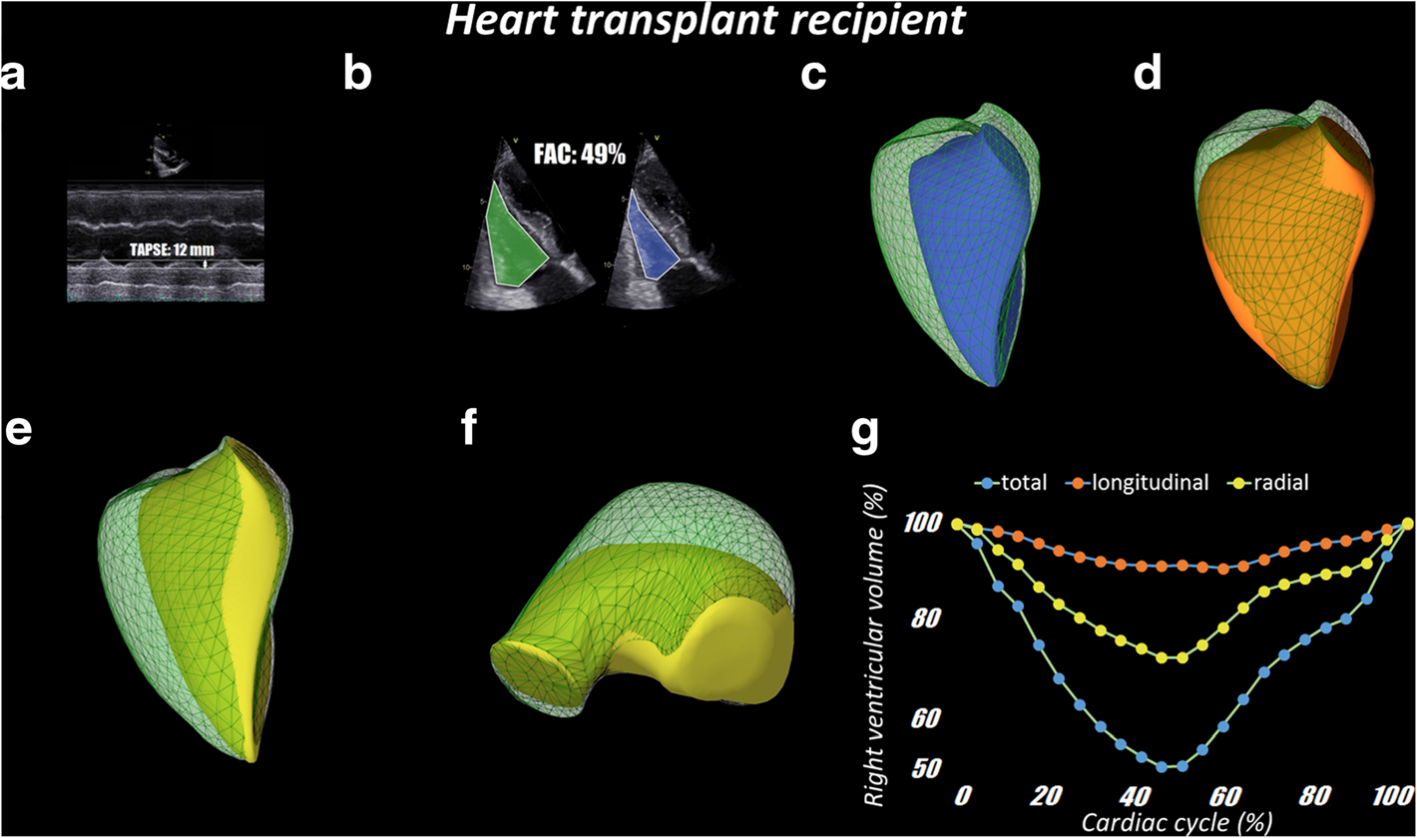
Representative heart transplant recipient. Panel **a**: TAPSE (12 mm) is reduced indicating moderate RV systolic dysfunction. Panel **b**: FAC (49%) is preserved indicating normal RV systolic function. Panel **c**: RV end-diastolic (90 ml) and end-systolic (44 ml) volumes as well as RV ejection fraction (51%) obtained from 3D echocardiography data sets show normal RV systolic function. Panel D: RV longitudinal displacement is markedly reduced. Panel **e** and **f**: By removing the longitudinal and anteroposterior components of RV volume change allows the appreciation of the increased extent of radial displacement of RV wall. Panel **g**: Quantitative analysis of the relative contribution of longitudinal and radial RV wall displacement to global RV volume change confirms the significant reduction of longitudinal displacement and an increase of radial displacement as the main mechanism to preserve RV stroke volume and ejection fraction (patient 144 days after the transplantation)

**Fig. 8:**
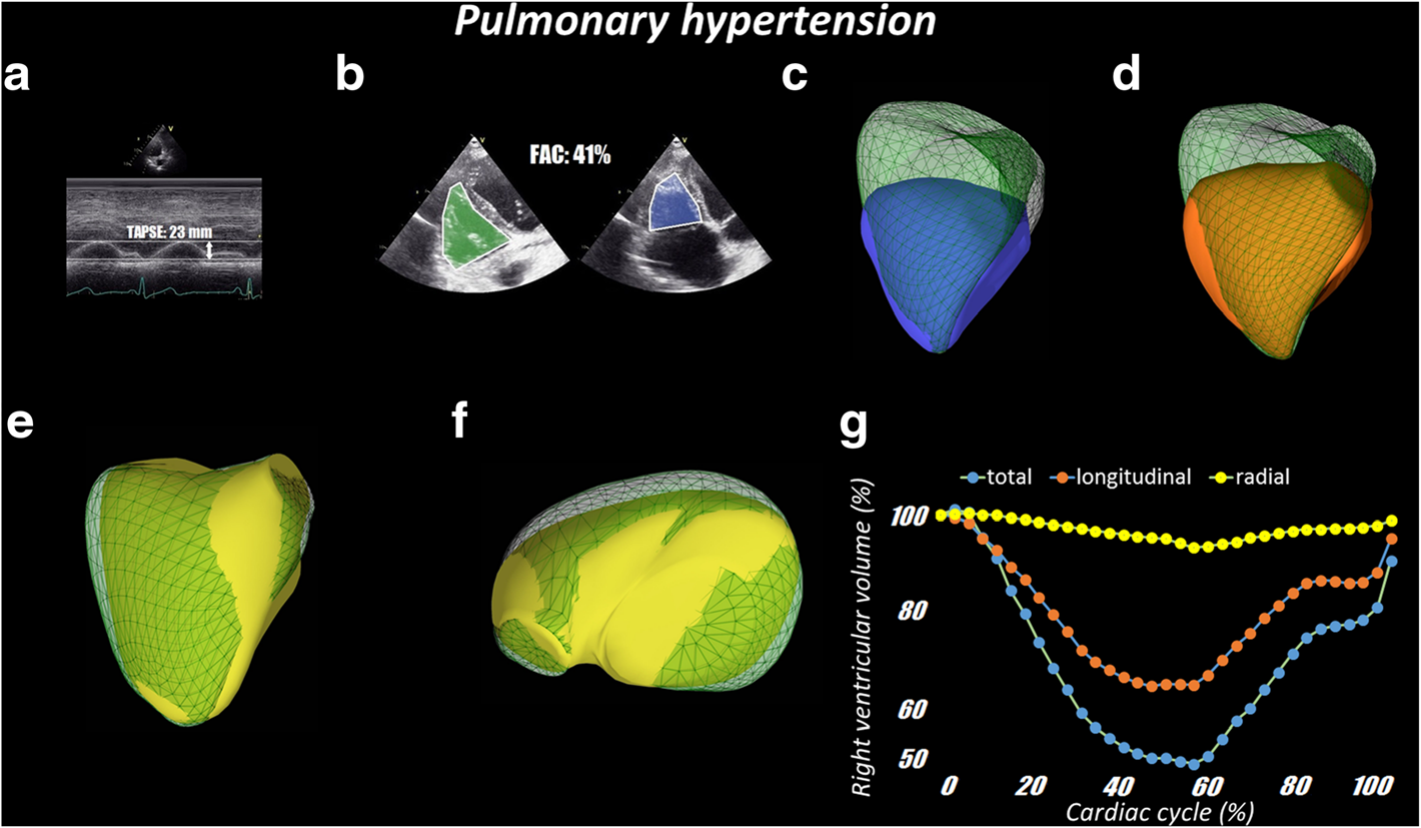
Representative pulmonary hypertension patient. Panel **a**: TAPSE (23 mm) was normal. Panel **b**: FAC (41%) was also normal indicating preserved RV systolic function. Panel **c**: RV end-diastolic (92 ml) and end-systolic (48 ml) volumes as well as RV ejection fraction (48%) obtained from 3D echocardiography data sets showed normal RV systolic function. Panel **d**: the longitudinal displacement appears supernormal. Panel **e** and **f**: By removing the longitudinal and anteroposterior components of RV volume change, the radial displacement of RV wall appears dramatically reduced. Panel **g**: Quantitative analysis of the relative contribution of longitudinal and radial RV wall displacement to global RV volume change confirms the significant reduction of radial displacement and an increase of longitudinal displacement as the main mechanism to preserve RV stroke volume and ejection fraction in chronic, compensated RV pressure overload pathophysiology (pulmonary artery systolic pressure: 69 mmHg)

Using 3D echocardiographic datasets, RV volumes and global ejection fraction can be measured with a good correlation with MRI results [8, 9]. However, we still lack data about the relative contribution of longitudinal, radial and anteroposterior component of RV wall motion to global ejection. During physiological conditions, the longitudinal shortening was suggested to account for the majority of RV pump function, however, larger normative studies are needed to characterize potential age and gender related alterations [3]. Moreover, there are several diseases and clinical scenarios, where the normal ratio between the different mechanisms can change. Clinical interest includes post-cardiac surgery patients, heart transplanted patients (with evidently reduced TAPSE values without signs and symptoms of right heart failure; Fig. 7), pulmonary hypertension patients (with often severe symptoms and dilated RV along with preserved TAPSE; Fig. 8), congenital heart diseases, elite athletes, etc. Evidence suggests that RV longitudinal component measured by TAPSE is markedly reduced in post-cardiac surgery patients despite the absence of global RV ejection fraction decrease [17]. Recent studies with MRI suggest that RV function is reflected better by radial rather than longitudinal wall displacement in pulmonary hypertension patients [18, 19]. Pettersen and coworkers demonstrated that, after the Senning procedure (which transforms the RV to the systemic pump of the circulation), the patients have more pronounced radial component of RV function [20]. As a special case of RV overload, physiological or even detrimental effects of regular and/or acute exhaustive physical exercise may be of clinical interest [21, 22]. After an endurance race, conventional parameters suggest a less pronounced decrease of longitudinal shortening compared to ejection fraction, which also refers to a post-race functional shift [23]. Moreover, there is a growing interest in separated evaluation of longitudinal and radial RV function in pediatric cardiology patients [24]. These studies have shown the importance of the varying contribution of longitudinal and radial component in certain conditions, but did not provide distinct quantitative assessment for these phenomena. The ReVISION method may be the first to answer these questions by quantifying all major mechanisms using a single 3D dataset obtained by the easily accessible and non-invasive transthoracic echocardiography.

### Rationale of the functional shift

The underlying causes of the varying relative contribution of these mechanisms to global RV function in different cardiac conditions remain to be clarified. Beyond the mechanical effects of volume and/or pressure overload, there are several other and often overlapping factors that may be responsible for these findings. Several research groups hypothesized that the myocardial fiber architecture of the RV can change in certain conditions. Classical anatomical dissections revealed that in patients with congenital heart malformations such as Tetralogy of Fallot, pulmonary or tricuspid atresia, significant changes can be observed in the myocardial fiber arrangement within the RV wall. These changes include the more oblique orientation of the longitudinal layer, and also the presence of a middle circumferential layer which is absent from physiological conditions [25, 26]. Pressure overload has been proven to induce changes in RV fiber orientation as well, with a relative dominance of the circumferential fiber direction within the RV wall [27]. These effects may manifest in changes of RV shape, which can be severely deformed in pulmonary hypertension patients [28] and may also induce functional remodeling. Radial shortening, which is usually generated by the circumferential fibers in the subepicardial layer of the free wall, may reflect RV pump function better in this scenario than longitudinal shortening [18]. Considering that RV function has shown to be a powerful predictor of the prognosis of pulmonary hypertension [29], the possibility of a distinct quantification of the radial and longitudinal contribution to global RV ejection fraction may provide valuable information about the early detection of impaired RV myocardial function (e.g. when global ejection fraction is still normal) and RV functional remodeling during the follow-up of these patients. Moreover, there are very limited data on the subclinical stages of the disease, and distinct quantification of the radial and longitudinal contribution to global RV ejection fraction may serve as a screening method to detect initial RV involvement in patients with systemic sclerosis, idiopathic pulmonary fibrosis, etc. The function of the interventricular septum accounts for a significant part of global RV ejection fraction, mainly by its longitudinal shortening [3]. Some authors emphasize the detrimental effect of interventricular septal abnormal motion, which occurs in cardiothoracic operations that involve cardiopulmonary bypass [30, 31]. Pericardial constraint and ventricular interdependence can be hampered by the opening of the pericardial sac, which may result in RV functional alterations [32, 33]. However, left ventricular contraction contributes to RV function also by stretching the RV free wall over the interventricular septum. Importantly, this component may be at least partly characterized by the third (e.g. the anteroposterior) component of RV wall motion. No data exist on the functional significance of RV wall anteroposterior motion, which can be quantified by our novel method as well. Loss of innervation may be another important factor which may affect global RV function in patients undergoing heart transplantation. Evidence suggests that the transplanted heart can be reinnervated by both sympathetic and parasympathetic fibers [34, 35], and the re-gain of autonomic nervous control can result in the recovery of RV longitudinal shortening over time.

### ∆*V*/∆*t analysis*

Current literature is scarce about the clinical value of RV peak ejection rate and peak filling rate due to their previously complicated measurements with radionuclide ventriculography [36, 37]. 3D echocardiography offers an easier way to measure them, allowing also the decomposition of the different contributions of the different RV wall motion directions that may provide additional physiological and pathological insights into the systolic and also diastolic dynamics of the RV. Considering that the fiber orientation of the RV varies in different regions and also transmurally [1], this method may be a marker of heterogeneous contraction and relaxation pattern in certain cardiovascular conditions.

## Conclusions

Since the possibility to quantify the relative contribution of the different components of right ventricular wall motion to global right ventricular ejection fraction would be of high clinical interest in both physiological and pathological cardiac conditions, we developed the ReVISION method to obtain these components separately from 3D data sets of the right ventricle by echocardiography. Since there is no reference method to test the accuracy of our measurements, only prospective outcome studies can assess the clinical value of our approach.

The ReVISION software can be freely downloaded for scientific purposes from www.revisionmethod.com
